# The cancer burden and cancer control in developing countries

**DOI:** 10.1186/1476-069X-10-S1-S2

**Published:** 2011-04-05

**Authors:** Paola Pisani

**Affiliations:** 1 University of Turin, Italy

## Abstract

Substantial changes in large parts of the developing world have materialised in the last three decades. These are extremely diverse countries with respect to culture, societal values and political arrangements, but sharing one feature - prevalent poverty and limited resources to protect the health of individuals. The control of emerging chronic diseases in low-resource countries is a formidable challenge. For this reason any intervention should be kept logistically simple and incorporated into a general plan aiming at building gradually the infrastructure that is necessary to bring care to the population at large. The present contribution summarizes some of the priorities in cancer prevention in developing countries and the underlying evidence base, and addresses some of the challenges.

## Background

Many countries in sub-Saharan Africa still struggle with endemic tuberculosis, malaria, AIDS, nutritional deficiencies and perinatal conditions that cause high rates of premature death and permanent disability, a disease burden at least one order of magnitude greater than cancer. But even where substantial economic development has taken place, as in Thailand, Malaysia, China, India or Brazil, it has failed to benefit society at large; rather, new health threats are on the rise with limited control of the long-term burden of prevalent diseases. Moreover, the lack of comprehensive planning of health systems has led to wider inequalities in access to health care.

Cancer control encompasses a package of diverse interventions [1] aiming at reducing morbidity and mortality from the disease, with wide variations in costs and potential impact. Under serious budgetary constrains and competition with the demands of other diseases, cancer control programmes need to make wise choices to maximise the efficacy of their investments.

**Figure 1:**
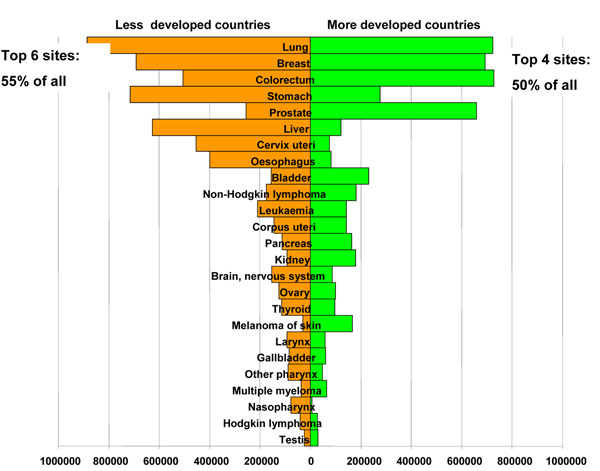
Cancer incidence 2008, males and females. Estimated numbers of new cases per year by level of economical development [2].

Choices should be driven primarily by the quantification of the problem combined with the feasibility and cost of different interventions. Means to monitor the occurrence of cancer in developing countries are still very limited, therefore planning relies largely on estimates. Based on the comprehensive GLOBOCAN2008 [2] dataset, in developing countries as a whole cancers of the lung, stomach, breast, liver, colorectum and cervix are the most common sites in this order, each is at least twice more frequent than the majority of any other cancer type (Figure 1); they are a mix of malignancies linked to infections and poverty (stomach, liver and cervix) and westernization of life styles (lung, breast and colorectum). The Figure 1 shows for comparison the ranking in affluent regions where the four top sites are lung, breast, colorectum and prostate. In the context of cancer control it is particularly important to remark that in both high- (generally rich) and low-risk (generally less wealthy) countries, such common sites account for only half of the burden.

## Options for prevention

Smoking of commercial cigarettes used to be uncommon in developing countries where tobacco smoking is a recent aspect of Westernization of life styles. While interventions to reduce the habit in rich countries is now showing positive results, the tobacco industry is pursuing new markets in the developing world [3,4]. Of all possible interventions to reduce the cancer burden, comprehensive programmes to prevent tobacco smoking are the most cost-effective: the impact on future chronic disease burden is the largest achievable by preventing one single factor, against measures that are inexpensive to governments such as banning smoking in public places and impose high taxes on the product. Tobacco prevention should be a priority for all countries.

**Figure 2:**
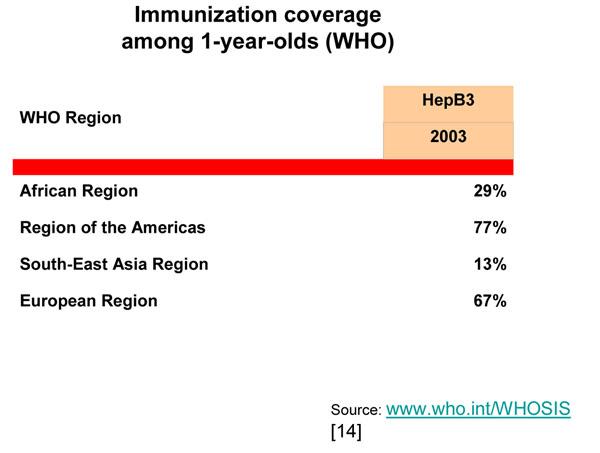
Immunization coverage among 1-year-olds (WHO)

Immunization of infants against hepatitis B virus (HBV) is probably the second most cost-effective option in regions where the infection is still endemic. Lorenzo Tomatis at the International Agency for Research on Cancer saw the potential of such public health measure in the early eighties when he promoted the establishment of The Gambia Hepatitis Intervention Study (GHIS). The main objective of the study was to prove the feasibility of such interventions and quantify the efficacy of immunization in preventing chronic liver diseases and hepatocellular cancer in an African country. Several more years of observation are needed to measure the full impact of the intervention; but high coverage and reduced incidence of chronic hepatitis have been achieved [5] promising success also with respect to the malignant disease. Sadly, immunization coverage remains low in the regions that would benefit most from this public health measure (Figure 2).

One of the most celebrated successes of cancer research is the recognition that virtually all cervix cancers are caused by certain types of the Human Papilloma Virus (HPV) with types 16 and 18 accounting for about 80% of the burden [6]. The finding led to the development and testing of vaccines that are now approved in many countries. The logistics of high coverage HPV immunization programmes is more complex than that of vaccination of infants since they target adolescents before they become sexually active. Inherent difficulties are amplified in developing countries where access to preventive medical services is limited in particular for girls. Even under the ideal (and unrealistic) hypothesis that all new generations of girls will be protected with vaccines, already infected women will continue to be threatened by cervix cancer for decades. A realistic view must recognise that large strata of women in developing countries will continue to be exposed to the infection and will develop the disease. Prevention of invasive cancer by early detection and timely treatment will remain therefore an important component of cancer control policies.

Compared with classical cytology-based screening, the HPV technology offers a valid and possibly more cost-effective strategy in secondary prevention of cervix cancer in low-resource settings, because the sensitivity and specificity of available HPV tests in exfoliated cells are much more reproducible than those of cytology which strongly depends on human expertise and skill [7]. In fact, to maintain high quality standards of screening by cytology in developing countries has proved difficult, costly, and too often ineffective.

In a large randomised trial in Kerala, India, Sankaranayan and colleagues [8] assessed the efficacy of a single examination by three screening modalities —HPV testing by Hybrid Capture II for 16 high-risk types, cytology and visual inspection with acetic acid (VIA) — in reducing mortality from cervix cancer compared with background rates achieved by usual care. The intervention was designed to maximise compliance of positive women with diagnostic follow-up and treatment, given local living conditions and infrastructure. Colposcopy and biopsy of all suspicious lesions were performed during the screening visit; small lesions were treated by cryotherapy or loop electrosurgical excision in primary health units. Eight years after a single screen examination, mortality from cervix cancer was halved in those HPV-tested. Smaller reductions were found in women screened by the other two modalities. This project proves that screening for HPV combined with the see-and-treat strategy is an effective intervention requiring limited expenditure.

There is no single strategy to develop cervix cancer control programmes from scratch. With a careful analysis of the size of the problem, feasibility, costs and expected outcomes against the background of existing infrastructure, plans can be gradually built from a minimal level —e.g. one life-time HPV-based screening test with timely treatment accessible to all women from age 35 years— and expanded on the medium- or long-term with immunization programmes and repeated screening. The condition for any intervention to be successful and cost-effective is to reach high coverage of the target population; therefore, much attention must be paid to the logistics of how the services are delivered in order to ensure access and high compliance.

Other preventive interventions that are the object of much research and activities in the West focus on nutritional habits and energy balance, clearly an increasing problem in emerging economies as shown by rising rates of diabetes [9] . Tackling obesity and excessive body weight is proving a difficult task in Western countries; it might be even more difficult in populations where over-nutrition coexists with malnutrition. Doctors, health care operators and teachers are in the best position to advise and counsel people; they should be made aware of their responsibility and be trained to monitor, educate and convey targeted messages.

The increased risk of breast cancer in emerging economies is seen as the direct expression of economical development; yet, our understanding of the modifiable causes of the disease is still very limited leaving little room for primary prevention beyond avoiding excessive body weight. Improving access to timely treatment of early palpable tumours is likely to result in a greater benefit to the population. Etiological research in populations still at low or intermediate risk for the disease offer instead powerful opportunities to test hypotheses based on observations made in the high-risk Western world.

Finally, an area that is often overlooked among preventive actions in low-resource countries is the uncontrolled use of carcinogens in industrial processes and economical activities, often imported from technologically advanced economies where regulations impose uses that are safe for workers and the environment, but less profitable. Any attempt to estimate the magnitude of the current and future disease burden due to potential carcinogens newly introduced under uncontrolled conditions would be highly controversial as lack of regulations implies also lack of monitoring of the amount, usage and disposal of hazardous substances. Nonetheless, whatever the size of the problem, ethical principles impose the inclusion in any cancer control programme of actions to prevent occupational exposure and environmental contamination with carcinogens. As a first step in this direction both rich and poor countries should be encouraged to sign up to the Rotterdam Convention whose objectives are to promote shared responsibility in the international trade of hazardous chemicals and to contribute to their sound use [10]. Bodies subscribing to the Convention commit to disseminate information on the characteristics and hazards of substances traded among parties.

## Cancer care

In rich countries the combination of early detection and new treatments that can improve disease outcomes have contributed to a modest but constant decline in cancer mortality rates that started in the 1980s [11]. The main determinant of such success is not the availability of more effective curative drugs but widespread access to health care through comprehensive health systems. In developing countries, poor infrastructure to intervene timely and to deliver even basic care may jeopardize well-meaning projects directed to specific diseases or conditions. Actions directed to specific outcomes, e.g. making treatment for some childhood malignancies and early breast cancer available to all cases, should be selected and included in a cancer control plans only if they have high potential impact (high rate of success) at affordable costs. Having this principles in mind, the development of means to provide pain relief and palliative care to cancer cases and other terminally ill patients is a much cost-effective option (in developing countries two cases in three die from the disease): to reduce the suffering is a benefit that all human beings value, it does not require sophisticated technology, and it is for all cases. Back in the 1980s, having recognised that lack of access to palliative care in poor countries was a major public health problem, the WHO established the Cancer Pain Programme [12]. Twenty years later, palliative care still struggles to gain priority with policy-makers. Only recently palliative care and pain relief programmes have been considered a necessary component of the minimum core services that health systems ought to offer to citizens [13] .

## Competing interests

The author declare no competing financial or non-financial interests.
